# Statistics for real-time deformability cytometry: Clustering, dimensionality reduction, and significance testing

**DOI:** 10.1063/1.5027197

**Published:** 2018-06-04

**Authors:** M. Herbig, A. Mietke, P. Müller, O. Otto

**Affiliations:** 1 Biotechnology Center, Center for Molecular and Cellular Bioengineering, Technische Universität Dresden, 01307 Dresden, Germany; 2 Max Planck Institute for the Physics of Complex Systems, 01187 Dresden, Germany; 3 Chair of Scientific Computing for Systems Biology, Faculty of Computer Science, Technische Universität Dresden, 01307 Dresden, Germany; 4 Center for Systems Biology Dresden, 01307 Dresden, Germany; 5 Zentrum für Innovationskompetenz: Humorale Immunreaktionen in kardiovaskulären Erkrankungen, Universität Greifswald, 17489 Greifswald, Germany

## Abstract

Real-time deformability (RT-DC) is a method for high-throughput mechanical and morphological phenotyping of cells in suspension. While analysis rates exceeding 1000 cells per second allow for a label-free characterization of complex biological samples, e.g., whole blood, data evaluation has so far been limited to a few geometrical and material parameters such as cell size, deformation, and elastic Young's modulus. But as a microscopy-based technology, RT-DC actually generates and yields multidimensional datasets that require automated and unbiased tools to obtain morphological and rheological cell information. Here, we present a statistical framework to shed light on this complex parameter space and to extract quantitative results under various experimental conditions. As model systems, we apply cell lines as well as primary cells and highlight more than 11 parameters that can be obtained from RT-DC data. These parameters are used to identify sub-populations in heterogeneous samples using Gaussian mixture models, to perform a dimensionality reduction using principal component analysis, and to quantify the statistical significance applying linear mixed models to datasets of multiple replicates.

## I. INTRODUCTION

The mechanical properties of cells are largely determined by the cytoskeleton,^1^ a polymer network that is not at thermal equilibrium and requires a continuous energy supply for maintenance. This polymer network and, hence, also the mechanical properties^2^ are subjected to alterations, for example, during cancer,^3–5^ differentiation,^6,7^ infection,^8^ or inflammation.^9^ Techniques such as atomic force microscopy (AFM),^10^ micropipette aspiration,^11^ optical stretcher,^12,13^ and optical tweezers^14^ have been used to gain insight into these alterations, but offer a relatively low throughput (<1 cell/s), which limits the total number of cells measured in an experiment. As a result, the datasets have a large variance and outliers are emphasized, while it is unclear whether these outliers belong to a sub-population (SP) of cells. Small datasets are also challenging when trying to avoid overfitting in machine learning algorithms, since the number of independent parameters in the model have to be smaller than the number of data points.^15^

These facts highlight the need for high-throughput measurement techniques for cell mechanical characterization. Larger sample sizes can be generated, for example, by using microconstriction arrays, which enable a throughput of approximately 3 cells/s.^9^ This technology obtains a mechanical readout of cells by pushing them through constrictions that are narrower than the cell nucleus. Microchannel resonators also use a tight constriction inside a microfluidic chip, which is placed on an oscillating cantilever. This allows measuring the passage time and also the buoyant mass of up to 200 cells/s by observing changes in the resonance frequency.^16^ In contrast, hydropipetting and deformability cytometry are microfluidic technologies that use wider constrictions and larger flow speeds to achieve contact-free stretching of cells by hydrodynamic forces at rates of up to 65 000 cells/s.^17,18^ For the duration of the experiment, the resulting data need to be stored on a camera, limiting this technique to a measurement time of a few seconds. Real-time deformability cytometry (RT-DC) utilizes a microfluidic system, where mechanical cell analysis is also based on hydrodynamic shear stress, but image acquisition and data evaluation is performed in real-time. This enables for characterization of arbitrary sample sizes with a throughput of up to 1000 cells/s and a direct data stream to a hard disc drive.^19^

The central element of RT-DC is a microfluidic chip that accommodates a channel, which is constricting the flow of suspended cells to a diameter modestly wider than the average cell size. In the channel, cells move with velocities on the order of 10 cm/s, and the parabolic flow profile induces shear and normal forces that are sufficient (≈1 *μ*N) to deform eukaryotic cells.^20^ With RT-DC, it is possible to measure several morphological and rheological properties of thousands of cells, yielding very robust information on the distribution of the sampled population. RT-DC has been used in numerous studies to measure changes in cell mechanics after drug treatment, infection, inflammation, cancer, gene knock-out, or during cell cycle and therefore demonstrated its importance in several fields of life sciences.^19,21–34^

Currently, RT-DC analysis is mainly based on the quantification of single parameters such as cell size and deformation and is limited to cells that are spherical in suspension.^19,30^ An analytical model allows extracting material parameters such as the elastic Young's modulus and was recently validated by numerical simulations.^20,35^ While this approach is sufficient to characterize homogeneous samples, it does not take advantage of the large number of parameters that can be extracted from image data. In this respect, a systematic statistical analysis of large RT-DC datasets is still lacking. Such a framework could be highly important, for example, to identify rare cells in heterogeneous populations without any external staining and could be used for label-free drug-screening assays.

Here, we introduce a set of relevant statistical methods and demonstrate their application to cell mechanical experiments. Gaussian mixture models (GMM) are implemented to identify a sub-population in mesenchymal stem cells based on biophysical properties.^23^ Additionally, we show that a simple dimensionality reduction using principal component analysis (PCA) is sufficient to discover a small very distinct sub-population in a retina sample, only using morphological and rheological information. With differential deformation, we introduce a novel parameter that takes into account the initial shape of the cells for data evaluation. This is highly relevant for label-free drug-screening assays that interfere with cell morphology, which hinders the use of standard deformation parameters that rely on a spherical reference shape.

For statistical testing, we define a linear mixed model that considers biological variation and reproducibility of an effect to compute significance. In an exemplary use case, we show differences in mechanical properties between the human osteosarcoma cell line MG-63 and skeletal stem cells (SSCs) and discuss biological implications for an improved understanding of mesenchymal stroma cells.^23^ The statistical test was implemented into an analysis software for RT-DC data with a graphical user interface.^36^

## II. MATERIALS AND METHODS

### A. Sample preparation

#### 1. Human osteosarcoma cell line (MG-63)

Dataset and methods for cell preparation have been published earlier.^23^ Briefly, the MG-63 human osteosarcoma cell line was cultured in Dulbecco Modified Eagle Medium with 10% fetal calf serum (Lonza, Basel, Switzerland), 100 U/ml penicillin, and 100 *μ*g/ml streptomycin at 37 °C. Only cells from passages 24 to 26 were used and a 70% confluence was assured by sub-culturing every 2–3 days. After trypsinization using a 0.025% (w/v) Trypsin-EDTA + 0.05% glucose solution (5 min at 37 °C), the cells were re-plated at a density of 2–$4\times 10^{4}$ cells/cm^2^.

#### 2. Human skeletal stem cells (SSC)

Dataset and methods for cell preparation have been published earlier.^23^ Briefly, the human skeletal stem cells were obtained during total hip replacement at Southampton General Hospital or the Spire Southampton Hospital in accordance with the Southampton and South West Hampshire Research Ethics Committee (Ref. No. 194/99/1 and 210/01). After extraction from the bone marrow and washing in α-MEM, the cells were filtered using a 40 *μ*m cell strainer and layered onto Lymphoprep™ (Stem Cell Technologies, Vancouver, Canada). A density gradient centrifugation was used to enrich and isolate the mononuclear cell fraction from the bone marrow, which is followed by labeling with a mouse hybridoma supernatant monoclonal (IgM) anti-human Stro-1 antibody. To isolate the SSC-enriched Stro-1+ fraction of cells, magnetic separation with anti-mouse IgM microbeads (Miltenyi Biotec, Bergisch Gladbach, Germany) was used.^37^ The enriched SSCs were incubated at 37 °C and 5% CO_2_ until confluence was reached in monolayer cultures in α-MEM supplemented with 10% fetal calf serum (Lonza, Basel, Switzerland), 100 U/ml penicillin, and 100 *μ*g/ml streptomycin.^23^ To bring cells into suspension for RT-DC, a 0.025% (w/v) Trypsin-EDTA + 0.05% glucose solution was used for 5 min at 37 °C. After centrifugation, cells were re-suspended in measurement buffer (MB) for RT-DC, which is based on phosphate buffered saline (PBS) and 0.5% (w/v) methyl cellulose (4000 cPs, Alfa Aesar, Karlsruhe, Germany).

#### 3. Retina cells

Retinal progenitor cells were isolated from Nrl-GFP (tagged with a green fluorescent protein) mouse lines at postnatal day ten. After dissection of the eyes, the retina was isolated and transferred to a papain solution (Worthington, Lakewood, USA) and incubated for 35 min at 37 °C.^38^ Retinas dissociated to a single cell suspension were centrifuged for 5 min at 300 rcf, re-suspended in Fluorescence-activated cell sorting buffer (2 mM EDTA and 1% bovine serum albumin in PBS) and passed through a 40 *μ*m nylon cell strainer (BD Biosciences, Heidelberg, Germany) before FAC-sorting. After sorting, the cells were centrifuged for 5 min at 300 rcf and re-suspended in the RT-DC measurement buffer.

#### 4. Human leukemia cell line (HL-60)

The dataset and methods for cell preparation have been published earlier.^30^ Briefly, the human peripheral promyelocytic leukemia cell line HL-60 was cultured in RPMI-1640 (Life Technologies, Carlsbad, USA) + 10% fetal bovine serum and 1% penicillin/streptavidin (Sigma-Aldrich, St. Louis, USA) at 37 °C and 5% CO_2_. 36 h after splitting, cells were centrifuged at 114 rcf for 5 min and re-suspended in the measurement buffer for RT-DC. Besides this control sample, also a *Cytochalasin* D (CytoD) treated sample was prepared by adding 1 *μ*M CytoD to the measurement buffer and incubation at 37 °C for 10 min.

### B. Experiment

**FIG. 1.**
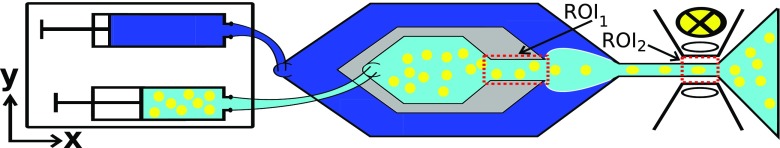
Sketch of the experimental setup consisting of two syringe pumps, providing a sample and a sheath flow. Cells are recorded in two regions of interest (ROI) inside the microfluidic chip. In ROI_1_ (“Reservoir”) cells move slowly and are subjected to nearly zero shear stress. ROI_2_ (“Channel”) is located within a narrow channel, where cells are deformed by hydrodynamic forces.

RT-DC experiments have been carried out as published earlier.^19^ Figure 1 provides an overview of the setup, which consists of a microfluidic chip assembled on an inverted microscope (Axiovert 200M, Zeiss, Oberkochen, Germany). The chip is made of polydimethylsiloxane (VWR, Darmstadt, Germany) and is connected to a syringe pump (NemeSyS, Cetoni, Korbußen, Germany) with two modules for driving the sheath (dark blue) and sample (light blue) flow along the central channel. Recording inside the chip is done using a high-speed CMOS camera (EoSens CL MC1362, Mikrotron, Unterschleißheim, Germany) and by illumination from a shuttered LED (Zellmechanik Dresden, Germany). The central squared constriction has typical diameters of 20 *μ*m × 20 *μ*m or 30 *μ*m × 30 *μ*m and a length of 300 *μ*m.

The measurement buffer (MB) for the experiments presented in this work is based on phosphate buffered saline (PBS) (Sigma-Aldrich, St. Louis, USA). We complement the MB with 0.5% (w/v) methyl cellulose (4000 cPs, Alfa Aesar, Karlsruhe, Germany), a biocompatible polymer, for density matching to avoid cell sedimentation and to increase the viscosity to obtain sufficient hydrodynamic forces in the microfluidic channel.^39^ The viscosity of the buffer is adjusted to 15 mPa s at vanishing shear rate. Shear-thinning effects lead to a reduced viscosity for elevated flow rates. For example, in a 30 *μ*m × 30 *μ*m channel, a flowrate of 0.16 *μ*l/s causes a drop in viscosity to 5.4 mPa s.^40^ In the evaluation of measurements, this is taken into account when extracting the Young's modulus *E* from RT-DC experiments.

The cells are deformed in the constriction (ROI_2_ in Fig. 1) by hydrodynamic shear forces, originating from a parabolic flow profile. The high-speed camera captures images of single cells inside the constriction zone, which are immediately analyzed to determine their contour, cross-sectional area [A (*μ*m^2^)], circularity [$C=2\sqrt{\pi A}/l;\;l$ – perimeter (*μ*m)], deformation (D=1−C), and other parameters (see Sec. II C). In addition to all obtained parameters, the contour and the bright field image of all measured cells are stored for further analysis.

### C. Parameters

**FIG. 2.**
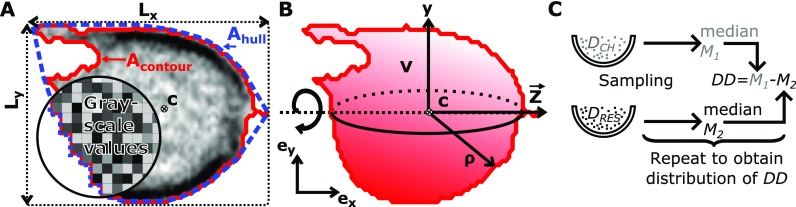
(a) Sketch indicating a cell and relevant cellular quantities. A bounding box defines the *x* and *y*-length. The area of the contour $A_{\text{contour}}$ determines the cell size and the area of the convex hull $A_{\text{hull}}$ is used to compute circularity. The average brightness and the Haralick features are computed using the grayscale values inside the contour. (b) The volume is computed by rotating the contour around z. The central axis z is given by the centroid *c* and the flow direction. (c) Differential deformation is calculated from samples within the reservoir $D_{\text{Res}}$ and the channel $D_{\text{Ch}}$ following a bootstrapping algorithm. Data resampling is done with replacement to construct a distribution representing the statistical difference between the reservoir and deformation inside the channel.

In the following, we introduce the parameters to analyze RT-DC data. These parameters are obtained from the contour and the bright field image of single cells [Fig. 2(a)], which are computed on-the-fly during data acquisition.

#### 1. Area A

The projected cell area defines the size of the cell and is calculated from a border following algorithm after image binarization [Fig. 2(a)].

#### 2. Volume V

A contour is a 2D projection of a cell and the volume can be determined under the assumption of rotational symmetry [see Fig. 2(b)], which is a valid assumption for cells that are not larger than approximately 90% of the channel width.^20^ Using the centroid *c* of the contour and the flow direction, we define a center axis z. Rotation of the contour is done by the following equation:^41,42^

$$V=\;\frac{1}{3}\oint_{\text{Contour}}x\left[xdy-ydx\right]=\;\frac{2\pi }{3}\int_{0}^{1}\left(\rho (s)\left[\rho \left(s\right)z^{\prime }\left(s\right)-z\left(s\right)\rho^{\prime }\left(s\right)\right]\right)\;ds,$$ (1)

with $\rho^{\prime }\left(s\right)=d\rho (s)/ds$ and $z^{\prime }\left(s\right)=dz(s)/ds$ and 0≤s≤1.

Here, the volume of a contour is expressed in *x* and *y* coordinates and then converted into an expression using ρ, referencing the distance from each point of the contour to the centroid and the coordinates along the central axis z. Since the contour is piecewise linear, the derivatives are just the differences between neighboring contour points.

For a contour in Cartesian coordinates *x* and *y*, the algorithm works as follows. The contour is shifted into the reference system of the centroid. First, only contour points below the center axis *z* are considered. Four volume parts are calculated for each contour point i using $v_{1,i}=d\rho *dx*\rho_{0}$, $v_{2,i}=2dx*\rho_{0}^{2}$, $v_{3,i}=\left(-1\right)*d\rho^{2}*dx$ and $v_{4,i}=\left(-2\right)*d\rho *\rho_{0}*x_{0}$, where $\rho =\sqrt{x^{2}+y^{2}}$ is the radius of each contour point and $x_{0}$ and ${\;\rho }_{0}$ denote the x coordinate and the radius of the first contour point, respectively. The final volume results from the sum of all contour point contributions and all volume parts:$\;V=\frac{\pi }{3}*\sum_{i}\left(v_{1,i}+v_{2,i}+v_{3,i}+v_{4,i}\right)$. The same routine is then applied for all contour points above the center axis *z* yielding a second volume estimation. Both volume estimates are averaged. This algorithm is implemented into the open source analysis software ShapeOut.^36^

#### 3. x-size L_x_ and y-size L_y_

A rectangular bounding box of cell major and minor axes defines its length in the flow direction $L_{x}$ and orthogonal to the flow direction $L_{y}$.

#### 4. Deformation D

The deformation measure D  describes how much the image of a cell deviates from a circular shape and is deduced from the circularity C as following: $D=1-C=1-2\sqrt{\pi A}/l$, where l is the perimeter of the cell and *A* its projected area. The perimeter is obtained from a convex hull of the original contour [Fig. 2(a)]. It is necessary to use the convex hull here, because non-convex, irregular shapes would lead to a significantly increased cell perimeter and thus an overestimation of the deformation. The area ratio *Δ* allows to exclude cells with non-convex shapes and is discussed in Subsection II C 8.

In addition, utilization of *D* for comparison between different samples also requires approximation of cells in suspension as spheres prior to deformation inside the constriction (ROI_2_ in Fig. 1). If this assumption is violated, differential deformation could be used.

#### 5. Differential deformation DD

Measuring the deformation only in the channel does not yield any information about the initial cell shape, which is essential to compare different samples if this shape deviates from a perfect sphere. A statistical information about the initial cell shape can be obtained from a reservoir (ROI_1_ in Fig. 1) measurement in front of the central constriction where cells move slowly and are subjected to vanishing shear stress. The idea of differential deformation DD is to subtract the reservoir from the channel deformation.

To determine *DD*, we employ a bootstrapping approach that subtracts statistical representations of reservoir from channel deformation values as sketched in Fig. 2(c). Thus, it compares two datasets: the deformations of one sample acquired in the channel (ROI_2_ in Fig. 1) $D_{\text{Ch}}\;$ and in the reservoir (ROI_1_ in Fig. 1) ${\;D}_{\text{Res}}$. From each vector of deformations values with length $n_{\text{ROI}}$ (where ROI represents the channel or reservoir measurement), sampling is done with replacement $n_{\text{ROI}}$ times. The resulting distribution is used to calculate a statistic like the median M. A single DD  value is then computed using ${\mathrm{DD}}_{j}=M_{\mathrm{j,Ch}}-M_{\mathrm{j,}\text{Res}}$. The process of sampling, calculation of M and ${DD}_{j}$ has to be repeated for a sufficient number of iterations (>1000) to obtain a bootstrap distribution of DD that follows a Gaussian distribution as a result of the central limit theorem. This distribution contains information about the difference in deformation between the reservoir and channel.

In contrast to *DD*, the relative deformation *RD* introduced earlier relates the observed difference in one sample, e.g., control measurement *M*_i_, to another sample, e.g., treatment measurement *M*_j_ by: $RD=\frac{M_{j,\text{Ch}}-M_{j,\text{Res}}}{M_{i,\text{Ch}}-M_{i,\text{Res}}}.$^30^ This parameter RD reduces the discrimination of two samples to a single number rendering statistical analysis challenging.

As differential deformation contains the information of the underlying sample distributions it can directly be used for statistical tests, e.g., linear mixed models, and can be computed for an arbitrary number of samples. Each sample yields a bootstrap distribution *DD* taking into account variations in the initial cell shape. This algorithm can, in principle, be extended to any parameter that requires comparing a state at vanishing shear stress with the shear-induced response of the sample. It is implemented into the open source analysis software ShapeOut.^36^

#### 6. Inertia ratio I and orientation φ

An additional measure of deformation can be obtained using the second moment of area. This value describes how area is distributed in space with respect to the centroid *c* [Fig. 2(b)].^43^ For a contour in Cartesian coordinates x  and y, the axial second moments of area are defined by

$$I_{xx}=\iint_{A}y^{2}dx\;dy,$$ (2)

$$I_{yy}=\iint_{A}x^{2}dx\;dy,$$ (3)

and the biaxial second moment of area reads

$$I_{xy}=-\iint_{A}xy\;dx\;dy.$$ (4)

We define the inertia ratio, *I*, as $I=I_{yy}/I_{xx}$, which expresses how much more a shape is elongated in direction ${\vec{e}}_{x}$ compared to ${\vec{e}}_{y}$ [Fig. 2(b)]. For a circle, I is equal to one and any elongation in direction ${\vec{e}}_{x}$ causes an increase in I. I is very robust with respect to pixelation effects (images of round objects appear discretized because of the pixel sensor of the camera) and shape irregularities. This implies that inertia ratio can be computed from the original shape and no convex hull is needed as it is the case for the computation of circularity [Fig. 2(a)].

The second moment of area also allows describing the angle φ  between the initial coordinate axes and the principal axes for the given contour. This angle quantifies the orientation of a cell with respect to the channel walls and can be calculated by^44^

$$\varphi =\;\frac{1}{2}\text{arctan}\;\left(\frac{2I_{xy}}{-\left(I_{yy}-I_{xx}\right)}\right).$$ (5)

#### 7. Elastic modulus E

Calculations of material properties from RT-DC measurements have been described earlier using an analytical approach based on steady-state hydrodynamics and linear elasticity theory.^20^ This approach has been validated by numerical simulations and it allows extracting the Young's modulus E based on circularity data (see Subsection II C 4).^35^

The Young's modulus E  is a material property, defined as the ratio between stress and strain in rheological experiments. In order to speed up the computation of E, a fine-meshed lookup-table was created, linking area and deformation to E.^40^

#### 8. Area ratio Δ

The ratio between the area of the convex hull of the contour and the area of the contour of the cell defines the area ratio $\Delta =A_{\text{hull}}/A_{\text{contour}}$. This value describes to which extend the calculated cell contour represents the actual cell shape and it can be used to describe its convexity and smoothness. The area ratio is large for very corrugated, non-convex contours and approaches one for smooth convex contours [Fig. 2(a)]. Its magnitude is essential to assess the range of validity when comparing samples and for extracting material properties from RT-DC experiments, since the underlying linear elastic models contain only convex shapes as a result of small deformations. In practice, only cells in a range from 1.0 ≤ Δ ≤ 1.05  are considered for an extraction of elastic moduli.

#### 9. Symmetry ratio S

The central axis z [see Fig. 2(b)] which is defined by the centroid of the cell and the flow direction is taken as a symmetry axis to split the contour in an upper and a lower part with the area $A_{\text{upper}}\;$ and $A_{\text{lower}}$, respectively. The symmetry ratio over the *x* axis

$$S_{x}=\;\frac{A_{\text{upper}}-A_{\text{lower}}}{A_{\text{upper}}+A_{\text{lower}}}$$ (6)

approaches 0 if the upper and lower part are equal in size. This symmetry parameter can also be defined for an axis orthogonal to z. This axis will point in direction ${\vec{e}}_{y}$ and result in $S_{y}$.

#### 10. Brightness B and standard deviation of brightness $B_{StD}$

Bright field images of cells can be used to obtain texture and transparency properties, which have been shown to be relevant for leukocyte detection and identification.^45–47^ Brightness identification is carried out within the contour defining a cell within a grey-scale-image [Fig. 2(a)]. An overall impression of the brightness of a cell is captured by the mean of all brightness values B, while the standard deviation $B_{StD}$ is a measure for the width of the distribution of grayscale values.

#### 11. Haralick texture features

The 13 Haralick features^48^ describe the texture of an image. They are based on the co-occurrence matrix, which describes how often a given gray scale value of a pixel is neighbored by a pixel with a certain gray scale value. The co-occurrence matrix of an image with *p* different grayscale values is p×p dimensional. The entry at position $\left(i,j\right)$ in this matrix shows how often the pixel value i is neighbored by the pixel value j. This calculation of the Haralick features is done using the gray scale values within the contour of the cell.

### D. Testing the distribution properties of cell parameters

A typical RT-DC dataset describes several morphological (area, brightness, Haralick texture features…) and rheological (deformation, inertia ratio, elastic modulus…) parameters of n measured cells. This can be represented by a n×k matrix, where k is the number of parameters. To test if a distribution of any parameter $d_{j}$ is distributed normally, the corresponding vector of length n is sorted in ascending order and quantiles m are computed according to Filliben's estimate

$$m\left(i\right)=1-0.5^{\frac{1}{n}},\;\text{for}\;i=1,$$ (7)

$$m\left(i\right)=\frac{i-0.3175}{n+0.635},\;\text{for}\;i=2,3,\ldots ,n-1,$$ (8)

$$m\left(i\right)=0.5^{\frac{1}{n}},\;\text{for}\;i=n.$$ (9)

A scatter plot showing the quantiles on the abscissa and the ordered values on the ordinate is called a probability plot. Gaussian behavior is indicated if the linear correlation coefficient between the ordered values and the quantiles is close to one. We use the square of the coefficient of determination ${\;R}^{2}$ to quantify linear correlation

$${\;R}^{2}=\;{\left(\frac{1}{n\sigma_{x}\sigma_{y}}\sum \left[\left(x_{i}-\mu_{x}\right)\left(y_{i}-\mu_{y}\right)\right]\right)}^{2},$$ (10)

with ${\;\mu }_{x}$, $\mu_{y}$ being the means of ordered values and quantiles, respectively, and $\sigma_{x}$, $\sigma_{y}$ the corresponding standard deviations.

### E. Gaussian mixture models *GMM*

Bayesian Gaussian mixture models (GMM) assume that a n×k dimensional matrix X can be described by a superposition of K normal distributions $N\left(\mu_{i}{,\Sigma }_{i}\right)$^49^

$$X\;∼\;\sum_{i=1}^{K}w_{i}N\left(X|\mu_{i}{,\Sigma }_{i}\right),$$ (11)

which are parameterized by a k dimensional vector of mean values $\mu_{i}$, a k×k dimensional covariance matrix $\Sigma_{i},\;$ and weights $w_{i}$ for each Gaussian i. The mean $\mu_{i}\;$ represents the point where the Gaussian distribution has the maximum probability and $\Sigma_{i}\;$ describes the spread of the distribution. Weights $w_{i}\;$ are scalars for every Gaussian, indicating its contribution to the mixture. The probability density function of a single Gaussian has the form

$$f\left(X|\mu_{i}{,\Sigma }_{i}\right)={\left(2\pi \right)}^{-\frac{k}{2}}{\left|\Sigma_{i}\right|}^{-\frac{1}{2}}\;\;\text{exp}\;\left(-\frac{1}{2}{\left(X-\mu_{i}\right)}^{T}\;\Sigma_{i}\;\left(X-\mu_{i}\right)\right).$$ (12)

The probability density function describes the probability that the given data X is in the range of values, expressed by a Gaussian with mean value $\mu_{i}$, and covariance matrix $\Sigma_{i}$. The parameters $w_{i}$, $\mu_{i}$, and $\Sigma_{i}$ are iteratively found using expectation maximation (EM).^50^ Algorithms to apply GMMs are available in the sklearn python package.^51^ We used the provided function with settings for unconstrained covariance matrices. Each event has a certain probability to belong to a given Gaussian distribution, and for clustering, events are assigned to the Gaussian where they have the highest probability.

### F. Bayesian information criterion *BIC*

For samples with an unknown number of sub-populations, the number of Gaussian distributions has to be estimated. Therefore, models with different numbers of Gaussian distributions need to be fitted and compared. We use the Bayesian information criterion BIC to quantify and compare the performance of different models to fit a given dataset. The BIC for a model with t parameters and sample size n reads^52^

$$BIC=\;-2\;\ln\left(L\right)+t\;\ln\left(n\right).$$ (13)

L  is the maximized value of the likelihood function p, found for a Gaussian mixture model with parameters μ,Σ,w

$$\ln\;p\left(X|\mu ,\Sigma ,w\right)=\;\sum_{j=1}^{n}\ln\left\{\sum_{i=1}^{K}w_{i}N\left(X_{j}|\mu_{i}{,\Sigma }_{i}\right)\right\}.$$ (14)

An increasing number of mixture components increases the degree of freedom of the model and will lead to higher likelihood values. The highest likelihood value can be obtained by fitting one Gaussian distribution to each event. To prevent such overfitting, the BIC  penalizes a high number of parameters t in the second term. This term increases with increasing n, which is in contrast to the Akaike information criterion (AIC).^53^ For this reason, the AIC tends to favor models with higher numbers of parameters t  compared to the BIC for datasets with large sample size n. Considering the aim to limit overfitting and as RT-DC data usually consists of several hundreds to thousands of data-points, we use the BIC in this paper throughout. The number of sub-populations in a heterogeneous dataset is found where the BIC reveals a minimum.

### G. Linear mixed models *LMM*

RT-DC as a label-free method can be used to test for the effects of specific compounds on the mechanical properties of cells. In such a setting, linear mixed models (LMM) can be used to estimate the statistical significance between datasets of multiple replicates.

Within the framework of LMM, a compound-specific effect on a parameter like deformation is called fixed effect β. Repeated measurements usually vary because of random effects u, which can arise, for example, from fluctuations in room temperature or because of biological reasons, like donor variability or passage time of the cells that are being analyzed. These effects are summarized in the following linear mixed model:

Y=Xβ+Zu+ϵ, (15)

where Y is the vector of the observed values, β is the fixed effect term, *u* the random effect term, and ϵ the random error, respectively. The experimental design like the number of biological replicates and treatment states are described by the design matrices X  and Z. Please note, while the random effect term *u* includes all systematic measurement bias, e.g., drift in temperature, random errors from unknown sources are included in the vector ϵ.

The R^54^ package “lme4”^55^ allows defining specific model properties such as number and dependencies of fixed effects β as well as random effects u. We specify a random effect term u with a random intercept to express the varying mean levels within the replicates of a parameter and a random slope to describe variations in the effect between pairs, e.g., control and treatment, of a specific replicate. Additionally, we define a so called “null model,” in which we drop the fixed effect term in Eq. (15).

The resulting models can be fit to the data, where lme4 uses a maximum likelihood or a restricted maximum likelihood method. The models return the maximized likelihood $L_{\text{Model}}\;$ and $L_{\text{NullModel}}$, respectively, which can be used to calculate the likelihood ratio $\Lambda =L_{\text{Model}}/L_{\text{NullModel}}$. The quantity Λ indicates how well a model can fit the data if it takes into account a fixed effect, compared to a model that does not. From this ratio, the p-value can be computed using Wilks theorem,^56^ which states that $-2\log\left(\Lambda \right)\;$ approaches a $\chi^{2}\;$ distribution. The resulting p-value indicates, if the null hypothesis (“both models are identical”) is true and it describes the significance of the fixed effect, since both models only differ by this term. Linear mixed models are implemented into the open source analysis software ShapeOut.^36^

## III. RESULTS

### A. Identifying universal distribution properties

**FIG. 3.**
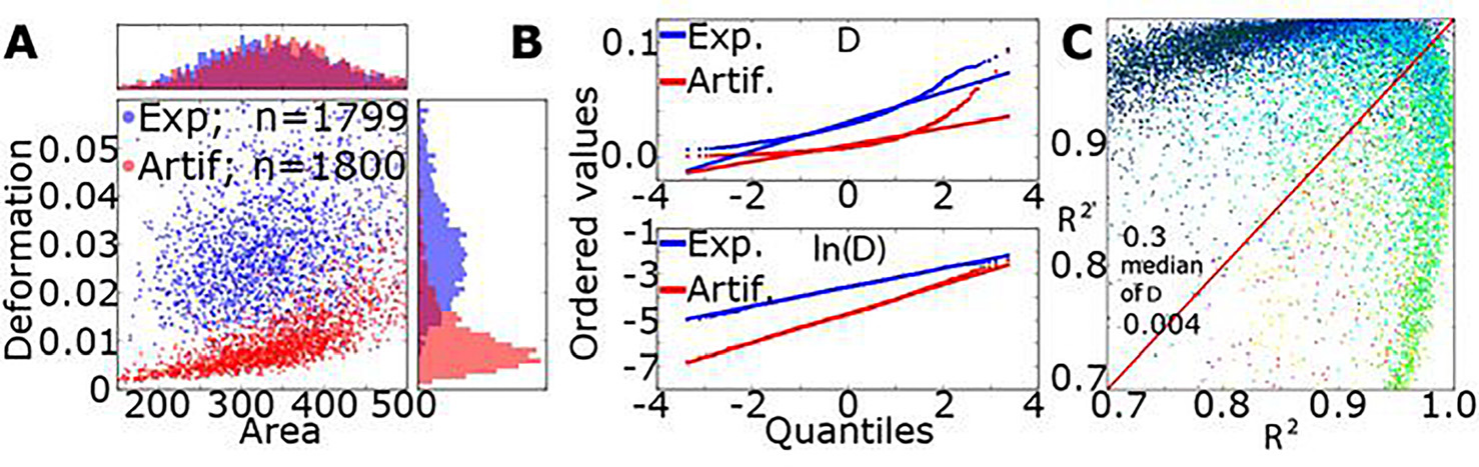
(a) Scatterplot of deformation vs. cross sectional area (*μ*m^2^) for an experiment (Exp, blue dots) with SSCs (using a flowrate of 0.16 *μ*l/s and a 30 *μ*m × 30 *μ*m wide channel geometry) and artificial data (Artif, red dots) derived from an analytical model. The histogram of area (projection on top) shows a Gaussian distribution, while the deformation histogram (projection on the right side) is skewed. (b) Probability plots of the deformation values of the experimental (Exp, blue dots and line) and the artificial dataset (Artif, red dots and line) of the original data (upper panel) and the log-transformed data (lower panel). (c) Coefficient of determination ${\;R}^{2}$ and ${\;R}^{2}\prime$, for the raw experimental deformation values and after a log-transformation, respectively. The dataset includes 14 862 experiments (26.25 × 10^6^ cells in total). A red line indicates $R^{2}=R^{2}\prime$ and the color code the distribution of the median deformation.

The measurable parameters of biological systems often dependent on aspects that always have certain variability during experiments, such as genetic and environmental factors. This intrinsic noise can be additive or multiplicative, typically resulting in a normal or log-normal distribution of the parameters it affects.^57^ A typical example of a deformation vs. area scatter plot derived from an RT-DC measurement of SSCs (flowrate 0.16 *μ*l/s in a 30 *μ*m × 30 *μ*m cross section) is displayed in Fig. 3(a) (blue data points). While the projection of cell area values in a histogram on top of the scatter plot suggest a normal behavior, the deformation values reveal a skewed distribution.

We can reconstruct the shape of the experimentally observed distribution qualitatively from an artificial dataset by applying the analytical model published earlier.^20^ Here, area A and elastic modulus E are assumed to follow a normal distribution (parameters: mean of area = 343 *μ*m^2^, std. dev. of area = 80 *μ*m^2^, mean of elastic modulus = 7 kPa, std. dev. of elastic modulus = 1.2 kPa, flowrate = 0.04 *μ*l/s, viscosity of medium = 15 mPa s, and channel width = 20 *μ*m × 20 *μ*m). The histogram projection of area and deformation for this artificial dataset follow the experimentally observed tendency [red data points in Fig. 3(a)]; thus, an elastic modulus *E*, which is normally distributed, translates into a skewed distribution for the deformation values *D* of the cells.

For further investigation of this observation, we first calculated the probability plot for all deformation values extracted from Fig. 3(a). The results are shown in Fig. 3(b), and a deviation from a Gaussian distribution (upper panel) is found for experimental as well as artificial data while a log-transformation of the deformation values results in a probability plot that can be well described by a linear function (lower panel).

The generality of the observation that deformation values follow a log-normal distribution is tested for more than 14 000 experiments by calculating $R^{2}\;$ and $R^{2^{\prime }}$ from Eq. (10). Here, $R^{2}\;$ and $R^{2^{\prime }}$ are the squares of the coefficient of determination for the original and the log-transformed data, respectively. Figure 3(c) indicates that for the majority of all experiments (11 344 ≙76.3%) ${\;R}^{2^{\prime }}>R^{2}$ and the data are closer to a log-normal distribution than to a Gaussian distribution. The analysis also reveals that experiments with small cell deformations (<0.07) follow a log-normal-distribution while measurements with a high median deformation (>0.07) are well described by a normal distribution [Fig. 3(c)]. The latter specifically include measurements of red blood cells, which are known to be very deformable.

The above analysis has been carried out for the majority of all parameters extracted from RT-DC measurements, e.g., area *A*, volume *V*, elastic modulus *E*, inertia ratio *I*, area ratio *Δ*, and cell orientation *φ*. It is remarkable that the majority of the assessed measurements follow a log-normal distribution for parameters that are related to cell mechanical properties ($D,\;I,\;L_{x},\;E$). This finding might originate from the fact that these parameters possess a lower/upper limit by construction, e.g., *D *=* *0. In RT-DC, experiments are usually carried out in the small deformation regime to remain within the validity of linear elasticity discussed above, and therefore, the resulting distribution will be skewed towards this lower/upper limit. Information on the underlying distribution representing the data is not only important for the validity of statistical tests (e.g., t-test) but also to characterize the dataset using model parameters (like mean and standard deviation) and to detect sub-populations, e.g., by Gaussian mixture models, assuming that all of them follow the same distribution.

### B. Detecting clustering using Gaussian mixture models *GMM*

**FIG. 4.**
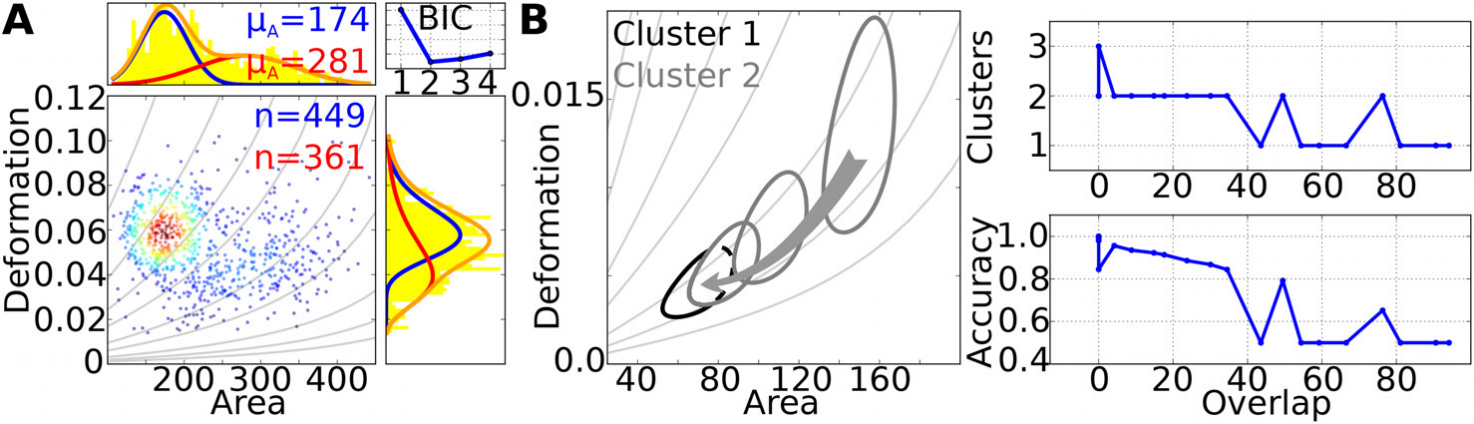
(a) Scatterplot of deformation vs. cell area (*μ*m^2^) of HL-60 and MG-63 cells mixed in a ratio of 1:1 and measured using RT-DC (flowrate of 0.32 *μ*l/s and a 30 *μ*m × 30 *μ*m wide channel geometry). 2D Gaussian mixture models with 1 to 4 clusters of sub-populations were fitted to the data and the BIC was calculated for each model (upper right panel). A model with 2 clusters shows a minimum in the BIC. The probability density function (PDF) of the corresponding model is illustrated in the histogram projections. The PDF of each cluster is indicated with a separate color (cluster 1, blue and cluster 2, red) and the superposition of both is plotted in orange. Gray lines represent isoelasticity lines, obtained from the analytical model. (b) The left panel shows 20% contour lines of two artificial datasets with identical means of elastic modules, but different means of area. Cluster 1 was stationary, while the mean size of cluster 2 was iteratively decreased, which results in an increasing overlap of the contours. In each iteration, a 2D-GMM was fit to the data and the BIC was used to predict the number of clusters. The upper right panel shows the number of clusters as function of contour overlap (%), while the lower right panel displays the accuracy of how many events were assigned to the correct population.

Mixture models are a powerful tool to separate and identify data belonging to different groups (clusters), e.g., to discriminate an unknown number of sub-populations in a larger sample. Following the previous section (Sec. III A), Gaussian mixture models (GMM) can directly be applied if the underlying mathematical distribution of the single clusters is known. To examine how well mixture modelling can distinguish two populations of cells inside a heterogeneous population, we prepared a biological sample of 50% HL-60 cells and 50% MG-63 cells, performed RT-DC measurements [Fig. 4(a)] and applied a two-dimensional Gaussian mixture model (2D-GMM) for area *A* and ${\text{log}}_{e}(D)$ to the data [projections in Fig. 4(a)]. A 2D-GMM with two Gaussians can be described using Eqs. (11) and (12) with K=k=2. The number of sub-populations (SP) is estimated using the BIC, which reaches a minimum for two clusters [Fig. 4(a), upper right panel].

The statistical information on the number of sub-populations is used to fit two normal distributions to the area and the deformation data after log-transformation. The result is shown in the projections of Fig. 4(a) revealing a superposition of two distributions (orange). Here, 55% of the cells were found to belong to the cluster of smaller cells (blue line, mean area = 174 ± 1.42 *μ*m^2^, mean deformation = 0.06 ± 5.6 × 10^−4^, error is the std. error of the mean) and the remaining 45% to the cluster of larger cells (red, mean area = 281 ± 3.37 *μ*m^2^, mean deformation = 0.045 ± 9.0 × 10^−4^, error is the std. error of the mean), which reflects closely the experimental conditions. Assuming that cells are spherical, we can approximate the diameter of the smaller cell size [blue line in Fig. 4(a)] to $d=2\sqrt{\text{Area}/\pi }=14.9\;\mu m$, which is close to the value expected for HL-60 cells.^58^ The population with larger cells reveals a diameter of approximately 18.91 μm, which refers to the typical cell size of MG-63 (Ref. 59) [red line in Fig. 4(a)].

For reference, HL-60 cells and MG-63 have been measured separately (supplementary Fig. S1). While mean values for deformation and cell size agree very well with the parameters extracted from the mixed sample, GMM detects two populations in each dataset. The low cell number in one sup-population might indicate outliers.

For assessing the performance of GMM with respect to the correct number of sub-populations and the correct identification of cells to each cluster, we created two artificial datasets with 1000 events, respectively. Both possessed a constant elastic modulus of *E *= 2.5 kPa but different mean cell area. While the position of the first distribution is kept constant at *A *= 70 *μ*m^2^, the mean area of the second one was reduced in steps of *ΔA *= 5 *μ*m^2^ until both populations started to overlap. Note, since we keep the elastic modulus of both distributions constant the second population moves along the isoelasticity lines when the mean cell area is reduced [curved grey lines, Fig. 4(b)].^20^

The overlap of the two populations was quantified from the corresponding probability density distribution following Eq. (12) and the resulting contour lines at the 20% maximum density [gray and black contour lines in Fig. 4(b)]. In each iteration of reducing the area of the second population, a 2D-GMM was fit to the data and the BIC was calculated to predict the number of clusters and to identify correct allocation of cells to each cluster.

Each contour encloses an area, which we name $a_{1}$ for the first contour and $a_{2}$ for the second. When the contours coincide, an area $a_{3}$ is enclosed by both contours simultaneously. The overlap is then defined by $\left(1-{{(a}_{1}-a_{3})/a}_{1}\right)*100\%$. Since deformation values have a different magnitude than cross sectional area values, the data were normalized by subtracting the mean and dividing by the standard deviation before computing the overlap.

We find that up to an overlap of approximately 35% the 2D-GMM plus BIC reveals the correct number of clusters [Fig. 4(b), upper right panel]. For performance quantification, the correct allocation of events to the sub-populations was checked by calculating the accuracy [Accuracy=(True positive+True negative)/Total number of events] in each iteration [Fig. 4(b), lower right panel]. For an overlap smaller than 35%, the 2D-GMM has an accuracy above 0.85. For an overlap of 35% and higher, GMM mostly fails to detect the second population and assigns all points of the second population to the first, which results in an error of 50%. The accuracy for GMM has peaks at 50% and 75% overlap, which shows that the accuracy can still be high if the correct number of clusters is detected. Therefore, an increase in accuracy can be expected if the number of sub-populations is known from complementary approaches.

Finally, we investigated the impact of cell number on GMM performance. We repeated the analysis described above with 30, 60, 125, 250, 500, 1000, 2000, 4000, and 5000 events in both sub-populations. We find that identification of clusters and correct assignment to a sub-population scales with the number of events highlighting the need of high-throughput analysis of large cell numbers (supplementary material, Fig. S2).

### C. PCA for multi-parameter analysis

Next, we would like assess the possibility of extracting information from multidimensional datasets of RT-DC experiments using principal component analysis (PCA). Dimensionality reduction using PCA has, for example, been applied to detect clusters in gene expression data and is based on the assumption that principal components with the largest eigenvalues obtained from the covariance matrix reflect best the structure of the clusters.^60–62^

**FIG. 5.**
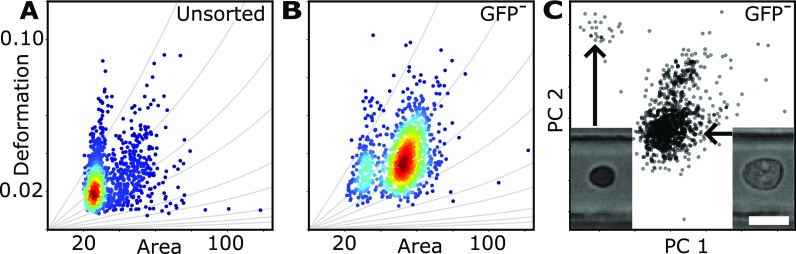
Scatterplot of deformation vs. projected cell area (*μ*m^2^) of (a) unsorted Nrl-GFP mice retina cells and (b) after depletion of GFP positive rod cells. (c) After projecting the multivariate data into 2D using PCA, a new population of small dark cells appears. Measurements have been carried out at flowrate of 0.04 *μ*l/s in a 20 *μ*m × 20 *μ*m wide channel geometry. Scale bar is 10 *μ*m.

Here, we use Nrl-GFP mice retina cells at postnatal day ten. At this maturation stage, many different cell types are present in the retina, but most of them are rod cells (≈80%). Performing RT-DC yields one homogeneous sub-population with large cell numbers around a projected cell area of approximately 20 *μ*m^2^ and a second heterogeneous sub-population around 40 *μ*m^2^ [Fig. 5(a)]. Depletion of GFP positive rod cells by FAC sorting leads to an enrichment in relative cell number of the second sub-population of larger cell size [Fig. 5(b)]. The final sample contains a certain number of rod cells as well as other types of retina cells and was measured using RT-DC.

By using PCA, we projected the multidimensional space of all RT-DC parameters introduced in Sec. II onto its principal components, uncovering a distribution of small and dark cells [Fig. 5(c) and supplementary Fig. S3]. This sub-population is very low in event number (1% to 3% of the total number of cells in the sample) indicating a rare cell type. The first principal component (PC1) has primarily contributions from the Haralick features, while the second principal component (PC2) is predominantly defined by contributions from area, volume, and brightness.

### D. Significance from linear mixed models *LMM* and likelihood ratio test

**FIG. 6.**
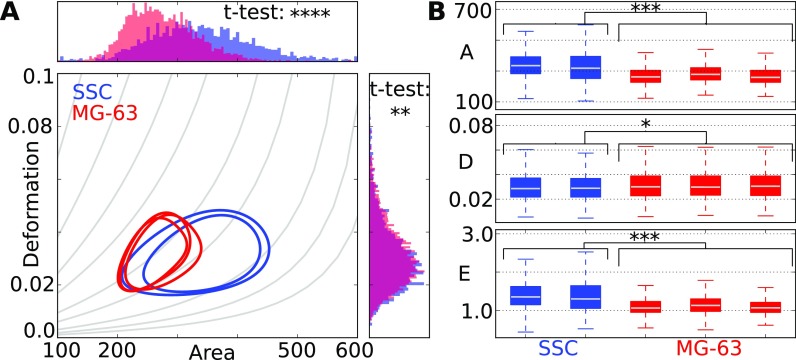
(a) Contour lines (at 50% of the maximum density) represent the distributions of two replicates of SSCs and a triplicate of MG-63 cells in area and deformation. The histograms show the distributions, after the data of replicates were merged. P-values are obtained from t-tests. (b) LMM analysis results in p-values for area ($p=2.15\times 10^{-4}$), deformation ($p=1.25\times 10^{-2}$), and elastic modulus ($p=2.10\times 10^{-4}$). The size of the boxes indicates the interquartile range IQR which spans from the first quartile $Q_{1}$ to the thirst quartile $Q_{3}$. The whiskers indicate the range from $Q_{1}-1.5\times IQR$ to $Q_{3}+1.5\times IQR$ and the line is the median. Measurements have been carried out at a flowrate of 0.16 *μ*l/s in a 30 *μ*m × 30 *μ*m wide channel geometry. (*p < 0.05, **p < 0.01, and ***p < 0.001).

The analysis of statistical significance is essential to compare single measurements and for data interpretation. The contour-plot in Fig. 6(a) shows 50% contour lines of a triplicate measurement of a human osteosarcoma cell line (MG-63, red) and a duplicate measurement of primary human skeletal stem cells (SSC, blue). MG-63 cells are used as a model system for SSC's. We quantified how similar both systems are in terms of cell mechanical properties. The histograms in Fig. 6(a) show distributions of area and deformation, which are partially overlapping. A t-test results in a p-value of $p=2.25\times 10^{-3}$ (deformation) and $p=7.09\times 10^{-219}$ (area) indicating a significant difference for both parameters. This surprising result originates from the features of the t-test, which renders small differences significant for larges sample sizes.^63^

Therefore, we would like to introduce an alternative to this standard test, which also considers the large sample size of RT-DC datasets. As cell deformation scales with cell size, the elastic modulus *E* will also be used to decouple both parameters before a statistical analysis is done. A boxplot confirms a reproducible variability in the mean levels of area A, deformation D, and elastic modulus E in-between the replicates [Fig. 6(b)].

For statistical data analysis, we designed a linear mixed model (LMM) that allows each replicate to have an individual mean and each pair of MG-63 and SSCs is considered by its individual difference. Note that the underlying dataset consists of two and three subsets, respectively. This incomplete pairing can occur especially when dealing with primary samples of limited availability and is included in the framework of LMM by managing such data sets using a maximum likelihood estimation.^64^ This statistical model can be applied to any parameter of the dataset. As Fig. 6(a) suggests, there is a large difference in mean area, which is confirmed by the significance level obtained from a likelihood ratio test based on the LMM (p<0.001). Also, the mean deformation D (p<0.05) and mean elastic modulus E (p<0.001) are significantly different [Fig. 6(b)], which leads to the conclusion that MG-63 (mean elastic modulus = 1.2 kPa) cells are softer than SSCs (mean elastic modulus = 1.5 kPa).

The analytical model, which is used for the transformation of the experimental data to the Young's modulus E is only applicable if a number of conditions is met. First, the cell deformation should not be larger than 0.03 in the channel to match the assumption of a linear elastic deformation regime made in the analytical model. Note that this limit can be increased, when using a numerical model to predict material parameters. Additionally, the cells need to be spherical prior to the application of stress. Information about the initial shape can be obtained from the reservoir measurement (ROI_1_ in Fig. 1). The assumption of spherical cells is met for most cells but not for adherent cells, for example, macrophages or activated neutrophils which look very irregular in the reservoir. In such cases, it is necessary to include the shape of the cells in the reservoir for analysis.

### E. Differential deformation

Currently, RT-DC does not allow to measure the same cell in the channel and in the reservoir, which would be important to track the deformation of cells that are not spherical. Here, we introduce differential deformation DD which uses the statistical difference of deformation values between reservoir and channel for data analysis as shown in Fig. 2(c).

**FIG. 7.**
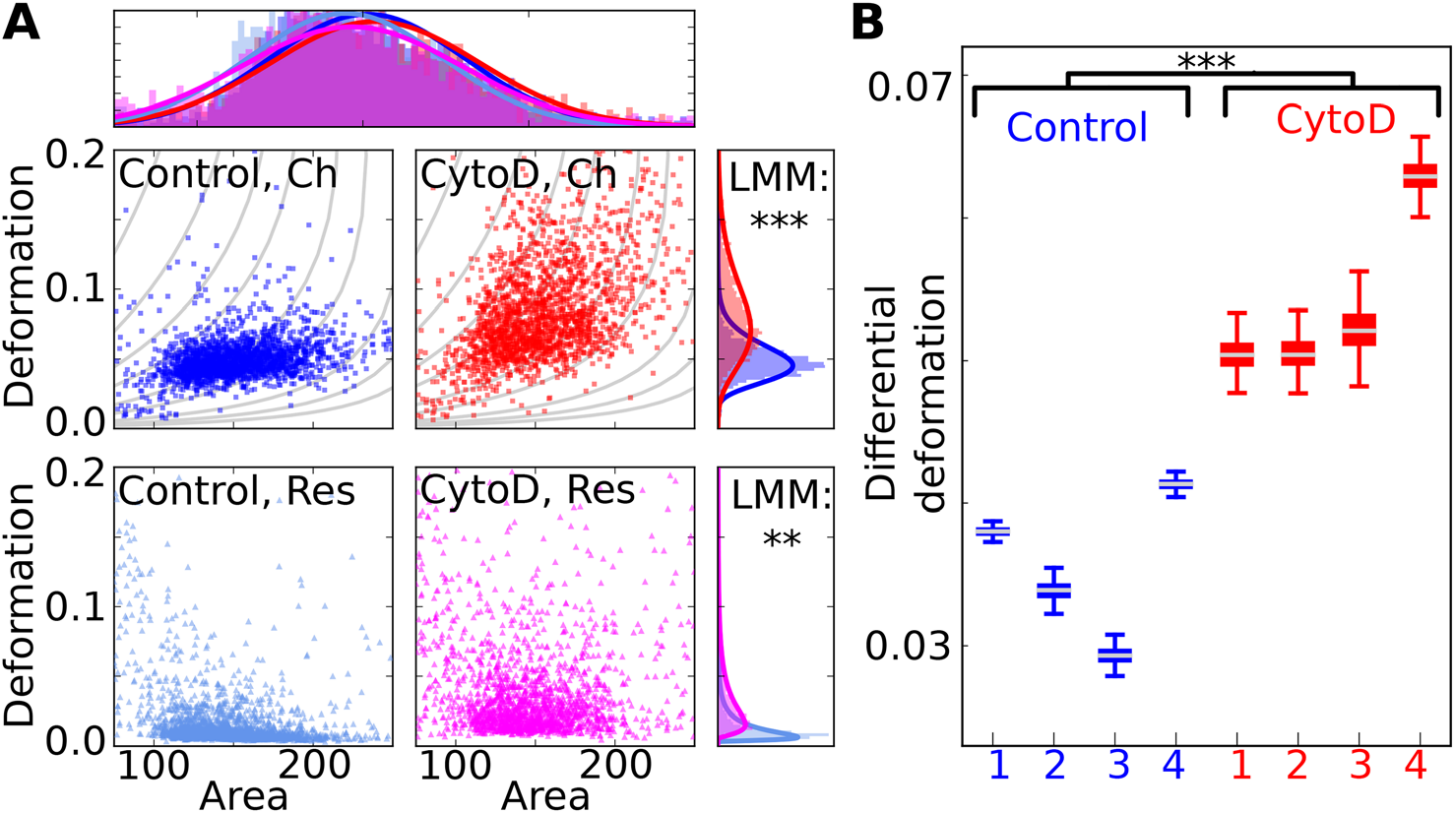
(a) Scatterplots of area (*μ*m^2^) and deformation show HL-60 cells, measured in the channel (top left, blue) and reservoir (lower left, light blue) and *Cytochalasin* D treated HL-60 cells in the channel (top right, red) and reservoir (lower right, magenta). The horizontal histogram shows that area distributions overlap. The upper vertical histogram shows that there is a difference in deformation in the channel and a LMM, applied to the dataset of four replicates results in a p value of $p=1.53\times 10^{-4}$. Also, the lower vertical histogram shows a significant difference in deformation of $p=1.58\times 10^{-3}$ (LMM) for experiments carried out in the reservoir. (b) Boxplot showing DD of four replicates of HL-60 (blue) and four replicates of CytoD treated HL-60 samples (red). LMM yields a statistical significant difference between the DD distributions ($p=5.01\times 10^{-4}$). Measurements have been carried out at a flowrate of 0.04 *μ*l/s and a 20 *μ*m × 20 *μ*m wide channel geometry. (**p < 0.01, ***p < 0.001).

Figure 7(a) shows representative scatterplots of area and deformation of untreated (Control) and 1 *μ*M *Cytochalasin* D (CytoD) treated HL-60 cells in the channel (top row of scatter plots) and in the reservoir (bottom row of scatterplots), where each dataset contains >1000 single cell measurements. The top histogram projection displays the area distributions of all measurements, which entirely overlap and are not significantly different in the reservoir (p=1.00, significance levels are obtained using LMM and the likelihood ratio test). Vertical histograms on the right show the deformation distribution for channel and reservoir, respectively. In the reservoir, there is already a significant difference (p<0.01) between the deformation distributions, which reveals differences in the cell shape between control cells and CytoD treated cells. The deformation, measured in the channel, is superposed by the initial shape and still shows a significant difference (p<0.001).

Figure 7(b) shows the boxplots for DD that were obtained for four control measurements and four CytoD treatments of HL-60 cells carried out at different days. Applying LMM to this data results in a p-value < 0.001, which indicates that CytoD has a significant softening effect when initial cell shape is considered for data evaluation. This result is in agreement with the original study where relative deformation was used for significance testing.^30^

## IV. DISCUSSION

In this work, we show that datasets from RT-DC experiments can be analyzed with a number of statistical methods to obtain new insights into morphological and mechanical properties of cell samples. First, we introduce a set of label-free parameters for the description of RT-DC data and study for each the underlying statistical distribution based on more than 14 000 datasets with more than 26 × 10^6^ cells in total. We demonstrate that deformation follows a log-normal distribution for datasets with a median deformation below 0.07, which is a threshold that is obeyed by the majority of all experiments. The log-normality of the deformation values can be understood as *D *=* *0 imposes a lower limit to the data. This result is also confirmed by artificial data obtained from an analytical approach published earlier.^20^ Higher median deformation values are observed, for example, for measurements of red blood cells. In this case, the datasets can be better described by a Gaussian distribution.

The knowledge about normality or log-normality is used in a Gaussian mixture model to decompose a dataset that contained contributions from two types of cells, human leukemia cells (HL-60) and human osteosarcoma cells (MG-63), but the principle can be extended to arbitrary numbers of subpopulations. Using the Bayesian information criterion, we have shown for this example that the correct number of clusters is found without user bias. Decomposition of a heterogeneous dataset into homogenous sub-populations is a crucial step before significance tests like linear mixed models can be applied. For an artificial dataset consisting of two populations we show that GMM predicts the number of clusters and correctly identifies cells with an accuracy of 0.85 until an overlap of 35% is reached. When the number of sub-populations is known from complementary methods, the overlap could increase to 50% while still reaching an accuracy of >0.6 for cell identification. In this assay, we also observe a random overestimation in the number of clusters found by the BIC based routine. This is a known problem that can originate from a non-Gaussian behavior of the underlying distributions. In such a case, a more flexible method such as Gaussian mixture copula models could be used.^65–67^ Also, the application of GMM is only valid for non-discrete distributions. Since some parameters of an RT-DC dataset are discrete, such as the pixel-precise length in *x* and *y* directions, this assumption has to be tested.

In a next step, we take advantage of the multi-dimensionality of RT-DC data and employ principal component analysis to screen for hidden sub-populations. Using retina cells isolated from mice we perform PCA on a dataset containing 11 label-free parameters and find a previously unknown fraction of small dark cells. This result hints towards pigmented cells that originate from the retinal pigment epithelium.^68^ Such a label free distinction of different retina cells is a promising result, which could be exploited to sort specific cells for transplantation purposes.^69^

We also introduce linear mixed models based on a likelihood ratio test for significance testing of replicate measurements. Standard approaches are likely to lead to statistical bias as the large sample size could render experimental noise significantly. We demonstrate LMM to provide robust statistical results while taking into account sample-to-sample variations. Using this method, we find that SSCs are significantly stiffer than the osteoblast-like MG-63 cells, showing that stiffness could serve as a marker for cell identification. This is in agreement with other studies, reporting that Mesenchymal stem cells are stiffer than osteoblasts.^70,71^

Formally, linear mixed models can reliably only be applied for data with normally distributed residuals. In practice, a deviation from this condition as observed in datasets following a skewed distribution has minor implications and gives an useful outcome.^72^ Improvement can be achieved using a generalized linear mixed model with a log-link function as an alternative.^73^ Another boundary condition is homoscedasticity, which implies equal variance of the residuals of the compared distributions. This condition cannot be granted for RT-DC data but a more robust implementation, which allows heteroscedasticity, has been published recently.^74^

Finally, we aim to improve the information content for main RT-DC parameters. Here, the elastic properties of cells can only be obtained within the assumptions of an analytical model or by a numerical approach. The analytical model does not consider feedback between deformation of the object and fluid flow inside the microfluidic channel, and deformation is limited to small values within the linear deformation regime. A numerical approach implementing this feedback has been published recently and permits a more precise determination of the elastic modulus for larger deformations.^35^ Both models are only valid for spherical cells, which is a good assumption for most cells in suspension but cannot be applied for most adherent cells. As an alternative approach to incorporate initial cell shape in the parameter analysis, we introduce differential deformation. While differential deformation enables a statistical comparison between cells before and during hydrodynamic stress exposure based on sample medians, this analysis does not integrate cells with different size distributions. Here, a numerical approach to predict elastic parameters for cells of arbitrary contour might be helpful but is computationally expensive when considering the variations in cell types.

In summary, we have explored the potential of RT-DC datasets for multi-dimensional data analysis. We present a number of statistical approaches that should be helpful for dimensionality reduction and significance tests while including the initial shape of the cell. In future, a combination of molecular markers based on fluorescence and label-free material properties based on cell mechanics might further increase the available information content.^75^ This might shed new light onto principles of how cells interact inside tissues.

## V. SUPPLEMENTARY MATERIAL

See supplementary material for additional data on Gaussian mixture models and principal component analysis.
